# Incorporating vertical movement of fishes in habitat use models

**DOI:** 10.1111/jfb.15857

**Published:** 2024-07-09

**Authors:** Ian A. Richter, Karen E. Smokorowski, Paul J. Blanchfield

**Affiliations:** ^1^ Fisheries and Oceans Canada, Ontario and Prairie Region, Freshwater Institute Winnipeg Manitoba Canada; ^2^ Fisheries and Oceans Canada, Ontario and Prairie Region, Great Lakes Laboratory for Fisheries and Aquatic Sciences Sault Ste. Marie Ontario Canada; ^3^ Department of Biology Queen's University Kingston Ontario Canada

**Keywords:** fish, fish habitat, habitat overlap, potential path volume, telemetry, white sucker

## Abstract

Fish telemetry studies now routinely collect positional and depth data, yet analytical approaches that integrate three‐dimensional data are limited. Here we apply the potential path volume (PPV) model, a method previously developed to estimate habitat volume based on rates of avian movement, to free‐swimming fish. Using a telemetry dataset of white sucker (*Catastomus commersonii*) from Turkey Lake (Ontario, Canada), we evaluated the effects of the number of spatial positions and different methods of selecting swim speed (v_swim_), a key parameter for PPV models, on habitat volume estimates. We subsequently compared habitat volume estimates and habitat overlap among white sucker pairs from the PPV models to those calculated using kernel utilization distribution‐based approaches. The number of spatial positions in the PPV model had a significant effect on habitat volume estimates, whereas the magnitude of the v_swim_ parameter or its specificity (constant value vs. fish‐season specific parameter values) did not affect habitat volume estimates. The PPV method resulted in larger habitat volume estimates and greater habitat overlap estimates among fish pairs relative to those obtained from a three‐dimensional kernel utilization distribution method. The PPV model is a useful analytical tool that, by incorporating potential animal movement into habitat use evaluations, can help answer key ecological questions and provide insight into fish space use in a wide range of conservation and management applications.

## 1 INTRODUCTION

Fishes are capable of making extensive three‐dimensional movements and exhibiting habitat occupancy patterns that may not be captured when data are analysed solely from a two‐dimensional perspective (Elliott et al., 2023; Matley et al., 2021). Rapid advancements in technology geared toward aquatic research have led to the development of improved telemetry systems capable of providing fine‐scale positioning data (Hussey et al., 2015) and collecting additional environmental data, such as pressure (i.e., depth), through different sensors (Cruz‐Font et al., 2019). Most fish telemetry studies have focused on two‐dimensional spatial habitat use (Guzzo et al., 2016; Ivanova et al., 2021; Rous et al., 2017; Watson et al., 2019), yet few have considered utilizing the depth data obtained from telemetry systems to investigate three‐dimensional animal movement and habitat use (Charles et al., 2017; Khosravifard et al., 2020; Simpfendorfer et al., 2012). Understanding how animals use their three‐dimensional habitat can provide valuable insight into their behavioral ecology. Numerous studies have investigated the spatial and vertical distributions of fishes separately (e.g., Blanchfield et al., 2009; Guzzo et al., 2016; Ketchum et al., 2014); however, this may lead to an incomplete understanding of habitat use. For example, animals may use various depths in different areas of their home range (Cott et al., 2015) and, therefore, will not have a consistent depth occupancy profile across space. Additionally, incorporating a third dimension to habitat movement will provide more accurate estimates for habitat overlap and potential interactions among individuals (Simpfendorfer et al., 2012). The evaluation of habitat overlap among fish pairs can determine whether individuals use, and possibly compete for, the available resources and habitat simultaneously. Telemetry systems can collect a vast amount of spatio‐temporal data on mobile animals, although many different analytical and visualization tools that can be applied to telemetry data remain unexplored.

A variety of analytical approaches are used to evaluate space use by animals, including kernel density estimation (Worton, 1989), convex hulls (Lichti and Swihart, 2011), and Brownian bridge‐based models (Aspillaga et al., 2019) among a wide range of options (Kraft et al., 2023). However, kernel density estimation is one of the more commonly used methods in telemetry studies (Guzzo et al., 2016; Ivanova et al., 2021; Matley et al., 2022; Watson et al., 2019). This non‐parametric approach involves calculating kernel utilization distributions (KUD) that represent the probability of finding an individual within a given area (Kraft et al., 2023). This approach is quite flexible, and different isopleths (e.g., 50% and 95%) generally represent different degrees of space used by the animal (Blanchfield et al., 2009). A potential issue with kernel density estimation is that every location is considered an independent observation (Fieberg, 2007). The independence of locations is likely not the case in telemetry projects, as the positional data of animals are inherently strongly associated with one another as animals are tracked across space and time (Boyce et al., 2010; Johnson et al., 2013; Rooney et al., 1998). If autocorrelation within the data is not addressed properly and conventional statistical analyses are used, the results could be biased as observations are not independent of one another, leading to, for example, the underestimation of home range size (Swihart & Slade, 1985).

Kernel density estimation can be extended to higher dimensional data and, therefore, can incorporate the vertical movement of organisms along a third dimension to evaluate the three‐dimensional (3D) habitat use of animals (Charles et al., 2017; Khosravifard et al., 2020; Simpfendorfer et al., 2012). An alternative to the kernel density estimation approach for evaluating three‐dimensional data is the potential path volume (PPV) model. The PPV was developed by Demšar and Long (2019) as an extension of the two‐dimensional potential path area (PPA) approach (Long & Nelson, 2012) that is commonly used in human mobility and transportation research to model accessibility (Patterson & Farber, 2015). The PPA and PPV focus on the possible movement of organisms between successive locations to determine the overall habitat that the animal could use. When a PPV is calculated, two locations are used as foci for an ellipsoid whose size is determined by the maximum speed the animal can travel and the difference in time between the two locations (Demšar & Long, 2019). The critical parameter for the model is the speed (v_max_ or v_swim_) at which the animal is capable of traveling, as this determines the longest possible path for the animal between the two successive positions within the given time period. One crucial data requirement for the PPV model is that the positional data are collected at a sufficiently fine temporal scale (Demšar & Long, 2019) to ensure that the resulting path volumes do not encompass the entire ecosystem due to long periods in which the animal's location is unknown. Similar to three‐dimensional kernel density estimation, the PPV approach can be used to investigate the use of the available 3D habitat by organisms. Although PPVs have been used to model avian movement (Demšar & Long, 2019), they have not been used to evaluate animal space use in aquatic ecosystems.

In this study, we explore the application of PPV models as a potential analytical tool to quantify the habitat use of freshwater fish within their three‐dimensional habitat. Here we use fine‐scale positional telemetry data from a population of acoustically tagged white sucker (*Catastomus commersonii*) in a small northern temperate lake. Although a wide range of characteristics can describe fish habitat use patterns, here we use the term fish habitat to describe the physical space usage of the fish. White sucker were tagged with pressure‐sensing transmitters that allowed for the collection of detailed 3D positional data over a 1‐year period. Specifically, we aimed to understand how the parameterization of v_swim_ and the number of positions affects the estimated habitat volume from PPV models. We compared habitat volume estimates from the PPV models to those obtained from a commonly used approach, kernel utilization distributions (KUDs). As habitat overlap is a focal point of animal space use studies (e.g., Bertolino et al., 2013; Madigan et al., 2022), we extended the PPV models of individual fish to assess whether habitat overlap among fish pairs differs among estimation methods and season.

## 2 METHODS

### 2.1 Ethics statement

The collection, handling, and surgical methods were approved by the Fisheries and Oceans Canada Animal Care Committee (protocol number: OPA‐ACC‐2022‐21; GWACC‐130—fish tagging protocol).

### 2.2 Study site and limnological data

**FIGURE 1:**
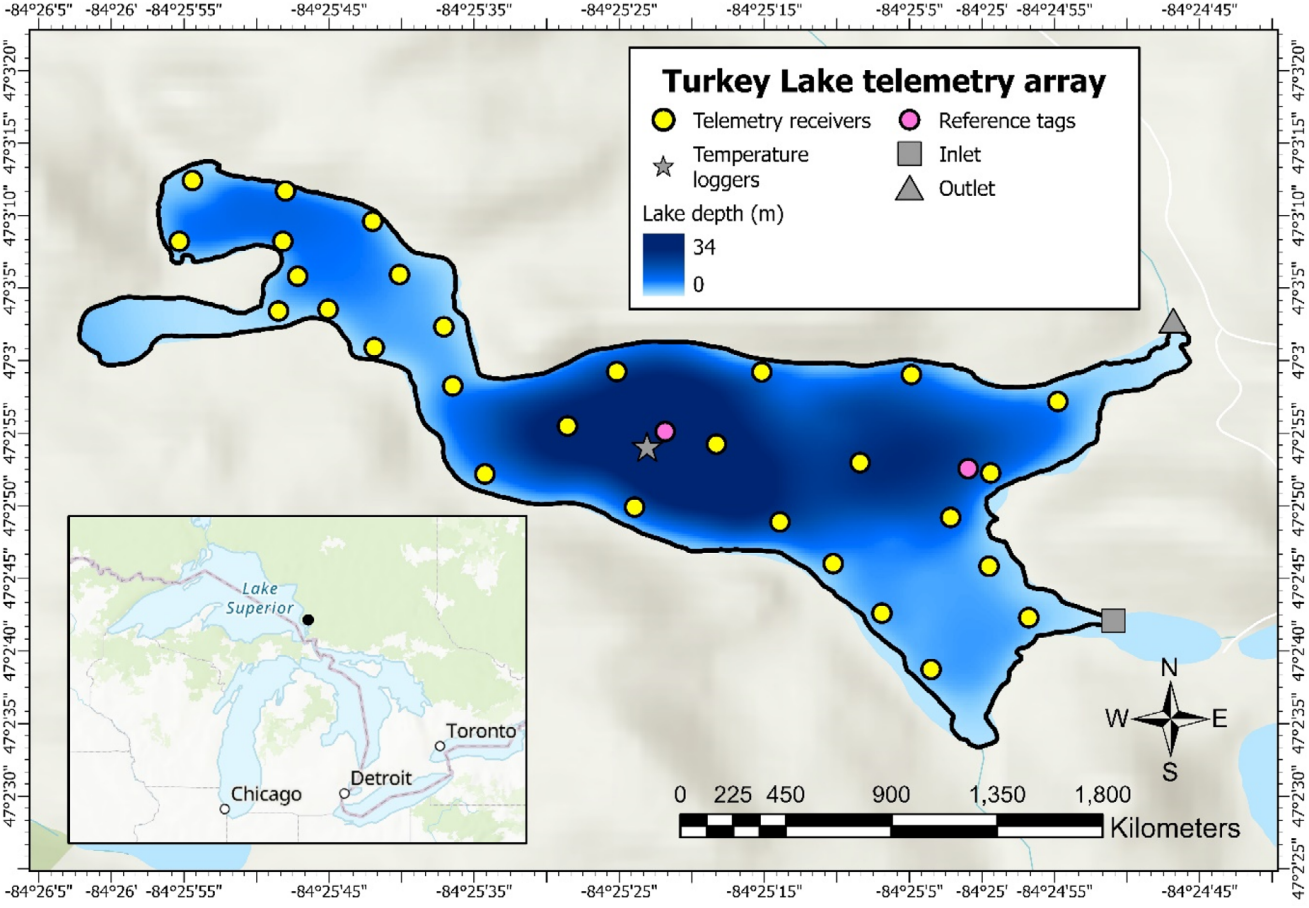
Map of the telemetry array in Turkey Lake, Ontario, Canada. A total of 29 VR2Tx acoustic receivers (yellow) and two reference tags (pink) were deployed throughout the lake. Water temperature profile data were collected from the temperature string (green star), located over the deepest part of the lake.

Our study site is Turkey Lake (84.42° W, 47.05° N), a 52‐ha lake on the Boreal Shield in the highlands east of Lake Superior and approximately 58 km north of Sault Ste. Marie, Ontario, Canada. The total habitat volume of Turkey Lake is 7,621,440 m^3^. The lake is situated in the Turkey Lakes watershed: a study area operated and used by various departments of the Canadian federal government since 1979 (Webster et al., 2021). The lake consists of a large primary basin with a smaller one in the northwest portion (Figure 1). Turkey Lake has a maximum depth of approximately 34 m with an average depth of 12 m. This oligotrophic lake is dimictic as it undergoes thermal stratification during the summer. We began collecting data at the study lake in October 2021, and this continued until October 2022.

The fish community of Turkey Lake consists of white sucker, lake trout (*Salvelinus namaycush*), brook trout (*Salvelinus fontinalis*), burbot (*Lota lota*), logperch (*Percina caprodes*), and various minnow species (e.g., northern redbelly dace *Chrosomus eos*, lake chub *Couesius plumbeus*, and emerald shiner *Notropis atherinoides*). White sucker are cool‐water benthivorous fish found in many aquatic habitats across Canada and the northwest of the United States and are often characterized as a generalist species that preys on primarily macroinvertebrates found in various habitats in lake ecosystems (Logan et al., 1991). White sucker are vital forage fish species that many recreationally and commercially valuable fish prey upon and can serve as essential ecological monitors of environmental changes and effects in specific systems (Arens et al., 2017; Oakes & Van Der Kraak, 2003). In Turkey Lake, white sucker are typically found on the bottom in the nearshore zone at depths <10 m and occupy similar areas of the lake in all seasons (Richter et al. in review).

We collected lake bathymetry data using a sonar unit (Humminbird Helix 7, ID #410590–1) mounted on a small boat, which we drove in transects across the lake. Transects were positioned 20–100 m apart depending on the settings of the unit (455 and 1200 kHz modes) to ensure that the transects were less than the range of the unit to ensure overlap across transects and full coverage of the entire lake. We used the ReefMaster software (https://reefmaster.com.au/) to develop a bathymetric map, with a resolution of 17 m, to determine the lake's physical boundaries and to be able to associate a lake depth with each fish position observed.

Temperature data were collected year‐round using an SDI‐12 temperature sensor string from Campbell Scientific (CS225‐L; https://www.campbellsci.ca/). The temperature sensor string was located at the deepest part of the central basin of Turkey Lake (Figure 1) and consisted of 18 temperature sensors, with the first sensor located 1 m below the lake's surface. After the first sensor, sensors were spaced at 1 m intervals until a depth of 12.5 m, at which depth the spacing between sensors increased to approximately 5 m intervals. Temperature data were collected every 10 min and were used to develop daily thermal profiles by linear interpolation. Seasons were delineated using daily mean surface water temperatures (<6 m depth), and the dates for ice‐on and ice‐off were based on daily images of ice conditions from trail cameras (Stealth Cam DS4K – STC‐DS4KTM) (Guzzo et al., 2017). We defined spring as the period after ice‐off and before surface water (defined as <6 m depth) temperatures exceeded 15°C. Summer was when the mean surface water temperature was greater than 15°C and fall occurred when mean surface water temperatures fell below 15°C until ice‐on. Winter was defined as the period when the lake was covered with ice (ice‐on to ice‐off ).

### 2.3 Fish capture and telemetry

White sucker were captured using trap nets in October 2021 when the surface water temperature was <15°C (i.e., spring and fall). Trap nets (1.2 and 1.8 m wide, 3.8 cm stretched mesh) were anchored to the shore at locations with a relatively shallow gradient and positioned perpendicular to the shoreline at a depth of 1.5 to 6 m. The trap nets were checked every 16–24 h across multiple days (2–3 days). A total of 10 white sucker (mean weight = 1257 g; range = 610–1700 g) were implanted with one of two different pressure‐sensing (depth) coded 69 kHz acoustic transmitters (V9P‐2x and V13P‐2x; see Table S1 for specifications) from Innovasea Systems Inc. (Bedford, Nova Scotia, Canada). The choice of transmitter was dependent on the size of the fish with all transmitters weighing <2% of the fish's body mass (Hubbard et al., 2021).

The surgical implantation of the transmitters generally followed protocols established in previous studies (see Blanchfield et al., 2005). Once captured, the fish were transported to a cooler filled with cold water from the lake, which was well oxygenated using a combination of a pump and an air stone. The fish were immobilized using Smith‐Root electric fish handling gloves (Tuononen et al., 2022; Ward et al., 2017), weighed (nearest gram; A&D model SK‐20KWP), and measured (to the nearest millimeter) for fork length and total length. Next, the fish were placed with their ventral side up between two high‐density foam pads on the surgery table to assist with immobilization. Water was circulated over the gills through the mouth using a pump to ensure constant well‐oxygenated water throughout the surgery. All surgical instruments were sterilized using betadine (10% povidone‐iodine), and lidocaine was injected subcutaneously at the incision site before surgery. An incision of approximately 20–30 mm, depending on the size of the transmitter, was made off‐center on the ventral side of the fish between the pectoral and pelvic fins to insert the transmitter in the body cavity. Depending on the incision size, one to three stitches using absorbable monofilament 3–0 diameter sutures (Ethicon coded Z761D) were needed to close the incision. Floy T‐bar anchor tags were inserted into the muscle on the dorsal side of the fish to allow for easy identification of tagged fish if recaptured. The fish were placed in another aerated cooler for observation and recovery post‐surgery and were released back into the lake once they displayed consistent normal swimming behavior (mean recovery time: ~18 min).

The fine‐scale positioning system consisted of 29 underwater omnidirectional acoustic receivers (VR2Tx, 69 kHz) deployed in October 2021 (Figure 1). Receivers were fixed to polysteel rope (length: 1–8 m, depending on lake depth and gradient) using zip ties and anchored to a cinder block via the same length of polysteel rope (see Figure S1 for receiver deployment diagram). The receivers were then located close to the anchors (1–4 m distance between the receiver and anchor) and were pointing upwards. A smaller, secondary anchor was attached to the cinder block using floating rope, which we used for receiver retrieval.

The receivers detect the acoustic signals emitted from the surgically implanted transmitters in white sucker and record the acoustic data (fish ID and depth) along with the date and time of detection. These data can then be used to solve for the position of the tagged individuals using trilateration methods focused on the time of the detections at the various receivers in the study system (Biesinger et al. 2013). VR2Tx receivers have built‐in sync tags to improve positioning accuracy and allow for internal clock synchronization during data processing. In addition, reference tags were deployed at two locations in Turkey Lake to improve positioning accuracy (Figure 1). The deployment of reference tags aids in identifying false detections and improves system performance for solving telemetry positions. Data collected from the reference tags were used to account for the spatial and temporal variability in the detection range of the telemetry receivers due to various environmental conditions (Brownscombe et al., 2019). Detection data were downloaded from the receivers in June 2022 and November 2022 and were used to solve fish positions by using the Fathom Position software by Innovasea Systems Inc. (https://www.innovasea.com/fish-tracking/products/fathom-software/), which has been used in other fine‐scale acoustic telemetry studies (Ridgway et al., 2023; van der Knaap et al., 2021).

Before analyses, the three‐dimensional white sucker positional data were subject to multiple filters. We excluded location data that were outside the study area based on the spatial (i.e., shoreline) and depth (i.e., lake depth + 2 m buffer) profiles of Turkey Lake. The position solver ensured that all the positional data did not occur at intervals shorter than the minimum time interval of successive transmissions (i.e., 120 or 240 s, depending on the transmitter model). We excluded positional data that occurred in fall 2021 to avoid possible unusual behavior associated with the surgery and focus on the fall 2022 to show the annual transition from summer to fall. The omission of the fall 2021 data from our analysis coincided with the removal of positional data related to a suspected fish mortality or emigrant (fish ID 8835, mortality date: November 11, 2021) based on a cease in detections. Data collected from fish 10195 were excluded from analysis after February 18, 2022, due to a lack of vertical and horizontal movements (>2 weeks); data collected from fish 8833 ceased after March 12, 2022. In addition, the hyperbolic positioning error (HPE), a measure of spatial uncertainty, was calculated for each position, and positions with an HPE > 20 were removed from the dataset (6.9%) (Rodrigues et al., 2022). The spatial details from the positional data were converted from latitude and longitude to Universal Transverse Mercator (UTM) to ensure that both spatial and depth data were measured in the same units (m) (Charles et al., 2017).

### 2.4 Parameterizing the PPV model

We calculated habitat volume using the PPV models at the daily (24 h) temporal scale for individual fish following the methods outlined in Demšar and Long (2019) and the *WildlifeTG* package in R (Demšar & Long, 2019; Long & Nelson, 2012). We calculated PPV for every individual path segment, defined as the straight‐line minimum distance between two consecutively observed positions, of an organism's trajectory and summed up these to produce a daily PPV. To calculate the PPV, the study area was discretized into 3D pixels known as voxels (m^3^) (Demšar & Long, 2019), and the PPV algorithm assesses whether each voxel is within the ellipsoid and subsequent PPV. For the daily PPVs, the individual voxels are assigned a binary value depending on whether they were included (1) or excluded (0) from the PPV encompassing all the individual path segments for a given day. To represent the frequency at which each habitat voxel is used by white sucker, we calculated the number of times an individual voxel was included in a PPV for each path segment within a given day rather than using a binary value for each voxel. Fish habitat volume was calculated as the product of voxel size and the number of voxels that had a value >1 indicating that the voxel was potentially used by the fish for the given day (Demšar & Long, 2019). We examine this modification to the initial model as an approach to assess the relative habitat use for all habitat voxels in the study area. The code for the base PPV model that we used, specifically the *ppv*() function, is openly available at https://github.com/udemsar/PPV.

The choice of voxel size and data filtering were essential considerations for calculating habitat volume using the PPV model. As tagged white sucker had greater horizontal movement rates (i.e., total distance traveled) relative to vertical movement, we rescaled the z dimension based on the ratio of horizontal and vertical movement rates (z_rescaled_ = z × 100) (Demšar & Long, 2019). The rescaling of the vertical dimension addresses the differences in horizontal and vertical movements of animals as the horizontal distance traveled is often considerably larger than the vertical distance traveled for most animals. The rescaling results in more voxels, with a smaller voxel size (voxel_z_ = voxel_length_/100), along the vertical profile of the study area to capture the vertical movements of fish at a finer scale relative to the broader horizontal movements of fish. Additionally, the rescaling of the vertical dimension reflects the differences in the horizontal and vertical confines of the defined study area such as a lake. Multiple values for the voxel length (5, 10, and 20 m resulting in voxel volumes of 1.25, 10, and 80 m^3^, respectively, after accounting for the rescaled vertical dimension) were considered, and we decided to use 20 m (V_voxel_ = 80 m^3^) in our analysis as this voxel length closely aligned with the resolution of the bathymetry data and there was no considerable difference in PPV habitat volume estimates among voxel sizes (Mean ± SD: 141,542 ± 265,944, 144,320 ± 284,606, and 148,083 ± 292,394 m^3^ for 5, 10, and 20 m v_length_ values, respectively).

The locations of the voxels were held constant across all scenarios using the spatial confines of the study area. The voxels were filtered to account for the physical barriers of the study area. Filtering the fish position data can result in extended periods between successive positions, and the temporal sampling resolution can influence the size of the PPV for individual path segments (Demšar & Long, 2019). Including path segments with large time intervals can significantly overestimate habitat volume as the resulting ellipsoid would encompass the others. Therefore, we omitted path segments, animal movements in between two observed positions, with a time interval that was three times greater than the temporal resolution of the fish telemetry data (1080 s) as suggested by Demšar and Long (2019). Through the omission of these path segments, we assumed that the PPV of these path segments was 0 m^3^ and subsequently did not contribute to the daily PPV estimate. This assumption would result in the underestimation of the daily PPV estimates in most cases but was applied to reduce the probability of overestimation by incorporating large, uncertain individual path segment PPVs to the daily PPV estimates.

A key parameter for the PPV model is the organism's maximum speed (v_max_), which determines how much habitat the organism could occupy between observed positions. As there are no defined approaches for selecting the v_max_ parameter value for PPV models for aquatic animals in confined study areas, we considered multiple different parameterization approaches to evaluate the effect of the v_max_ parameter value on the fish habitat volume estimates. Long and Nelson (2012) suggest calculating v_max_ using the two largest observed speed values from the observed telemetry data. Unfortunately, it is challenging to implement this approach for v_max_ calculations in small, confined study areas such as lakes because large v_max_ values result in the entire study area being included in the habitat volume estimates. The most important characteristic of the v_max_ parameter is its biological relevance to the study system being considered regardless of how the parameter value is calculated (Long & Nelson, 2012).

We estimated the v_max_ parameter for our PPV models using observed daily movement rates of white sucker in Turkey Lake. As we did not use the maximum speed value to parameterize the PPV models, we refer to the parameter as v_swim_ rather than v_max_ to differentiate our approach to PPV modeling approach from that used by Demšar and Long (2019) and Long and Nelson (2012). Daily movement rates were calculated as the total daily linear distance moved divided by the difference in time between the first and last observed positions for each date (daily v_swim_ range: [0.013, 0.158 m/s]). We calculated the daily movement speed for each fish‐date combination and selected v_swim_ parameter values based on the distributions of daily movement speed across all the fish. We arbitrarily selected the maximum speed value along with the daily speed values at the 50th, 75th, 95th, and 100th quantiles (Figure S2). As there was variation in the daily movement speeds among fish, we considered both v_swim_ parameter values specific to the individual fish and season combinations (see Figure S3 for fish‐season specific v_swim_ values used in model development) and those values from the distribution of movement speeds for all fish‐data combinations (v_Q50_ = 0.027 m/s, v_Q75_ = 0.039, and v_Q100_ = 0.158 m/s). Although these v_swim_ values are lower than other reported swimming speeds of white sucker (>0.5 m/s, Jones et al., 1974), these conservative values are more likely to reflect a sustained maximum speed across the temporal scale of our study rather than a singular burst of speed over a short period (Long & Nelson, 2012).

A few paths had observed speeds equal to or greater than the v_swim_ parameter, which can result in degenerate ellipsoids that are essentially a straight line between the two observed positions with a volume of 0 m^3^ as the ellipsoids will collapse into a simpler shape such as a straight line (Long & Nelson, 2012). Therefore, we considered an alternative maximum speed greater than or equal to v_swim_ for these specific paths to avoid this potential issue. We calculated the maximum speed for these specific paths as the sum of the observed speed and the difference between the observed speed and the assigned v_swim_ parameter value ($v_{\text{swim},i}=1.01v_{\mathrm{obs},i}+\left(v_{\mathrm{obs},i}-v_{\text{swim}}\;\right)$). This equation has been used to calculate v_swim_ in PPA and PPV models previously (Demšar & Long, 2019; Long & Nelson, 2012), and the multiplier ensures that the alternative maximum speed values will be greater than the observed speed for that path segment.

### 2.5 Habitat use models and statistical analysis

To understand how PPV estimates of habitat volume compare to commonly used methods, we also calculated kernel utilization distributions (KUDs) of white sucker from these same positional data. We considered two alternative scenarios to calculate habitat volume with the more common KUD approach. The first scenario involved calculating habitat volume from the KUD and the depth profile of the lake to reflect only having spatial data available from the tagged fish without any depth data. We assumed that the fish used the entire water column, and lake depth obtained from the lake bathymetry data was used for calculating the depth of fish habitat used. This approach was termed the 2D‐KUD method for estimating habitat volume. The second scenario incorporated the observed fish depth for each position into the estimation of the KUD to obtain habitat volume estimates (3D‐KUD method). We used the 95% isopleth for the KUD habitat volume estimates as this value is commonly used to represent the home range of animals (Heupel et al., 2004; Simpfendorfer et al., 2012). The *ks* package in R was used to parameterize the 2D‐ and 3D‐KUDs for fish at the daily temporal scale (Duong et al., 2022).

We calculated habitat overlap for each possible fish pair using the daily habitat volume estimates from the 2D‐KUD, 3D‐KUD, and PPV models. Habitat overlap between fish pairs was calculated as the number of overlapping voxels between the two habitat volumes multiplied by the size of the voxels.

Linear mixed‐effects models (LMMs) were used to evaluate the effect of the v_swim_ parameter and the number of positions on habitat volume estimates for the daily PPV models separately. Both models included log‐transformed habitat volume estimates as the response variable along with individual fish incorporated as a random effect. To investigate the effect of the v_max_ parameter on space use volume estimates, we included the v_swim_ quantile (Q50, Q75, Q95, and Q100) and the v_swim_ parameterization method (constant value across all fish or fish‐season specific values) as the fixed effects in the LMM. We evaluated the effect of the number of positions (n_positions_) on the space use volume estimates by parameterizing one LMM model that only included a linear term for n_positions_ and another LMM that included both a linear and quadratic term for n_positions_. The quadratic term was considered to address a potential bias toward larger daily PPVs with a lower number of daily positions. When there are more positions within the day, the contributions of individual path segments and the time difference between positions decrease with a larger number of daily positions, leading to smaller path segment PPVs that contribute to the overall daily PPV estimates. These models were compared to an intercept‐only LMM model that only included the random effect of individual fish as a predictor of habitat volume and no fixed effects to determine whether the fixed effects improved the overall model fit. AIC values were calculated for each model and used to compare model fit relative to the data. In addition, we calculated marginal and conditional variation explained (R^2^) for all the models (Nakagawa & Schielzeth, 2013). The LMMs were developed using the *lme4* R package (Bates et al., 2015) and evaluated using R^2^ values calculated using the *MuMIn* (Barton & Barton, 2019) package in R.

We used LMMs to test whether daily habitat volume estimates differed among methods (i.e., PPV, 3D‐KUD, and 2D‐KUD) and seasons. For the PPV models, we used median daily movement speed (Q50) from the fish‐season‐specific distributions for the v_swim_ parameter (Figure S3). Model selection, based on AIC values, was used to determine whether the method and season explained any variation in the habitat volume estimates and improved the model fit. Individual fish was the random effect once again. We parameterized the models with all possible combinations of the two fixed effects and their potential interaction (i.e., method, season, method:season) along with an intercept‐only model. The interaction term was included in the model selection process to determine whether the estimation method's effect on habitat volume differed among seasons as previous studies have observed seasonal patterns of habitat use in freshwater fish (Bloomfield et al., 2022; Blanchfield et al, 2023). Models were ranked based on their fit, which was measured using AIC values. Marginal and conditional R^2^ values were computed for each model.

We analysed the potential effects of season and estimation method on habitat overlap among fish pairs using a combination of two statistical models. The first model was a mixed‐effects logistic regression model that included a binary response variable that described whether the habitat of the fish pair overlapped (1) or did not overlap (0). This model was used to evaluate whether the probability of habitat overlapping between two fish is dependent on the season and the estimation method used to calculate fish habitat volume. Using only non‐zero estimates of fish habitat overlap, the second model was an LMM with the log‐transformed habitat overlap between fish pairs as the response variable. The purpose of this model was to quantify habitat overlap for fish pairs, using season and estimation method, in the presence of fish habitat overlap. Fixed and random effects were consistent across both models with season, estimation method, and an interaction term between the two variables as the fixed effects and fish pair as a random effect. Both models were compared to null models, which only included the random effects, using AIC values to determine whether season and estimation method significantly improved model performance.

## 3 RESULTS

**FIGURE 2:**
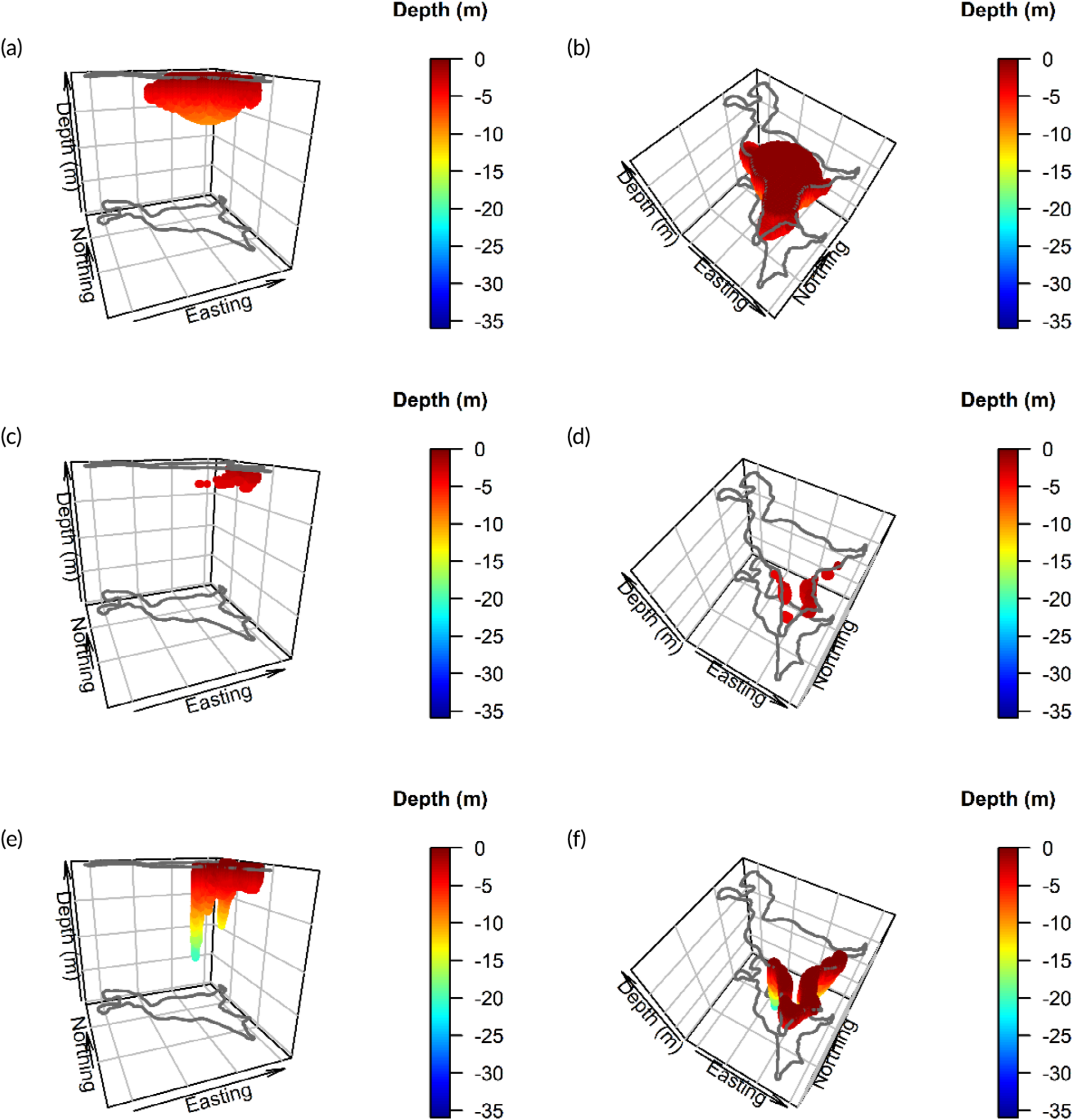
Comparison of the daily habitat use of an individual fish (ID: 3541) using the potential path volume (PPV) (panels a and b), 3D‐KUD (panels c and d), and 2D‐KUD (panels e and f) estimation methods from two different viewpoints. Positional data were collected on 2022‐08‐01. The color of the voxels indicates the depth of the lake. The gray outline represents the surface physical boundaries of Turkey Lake at the minimum and maximum depths (0 m and 34 m, respectively).

**FIGURE 3:**
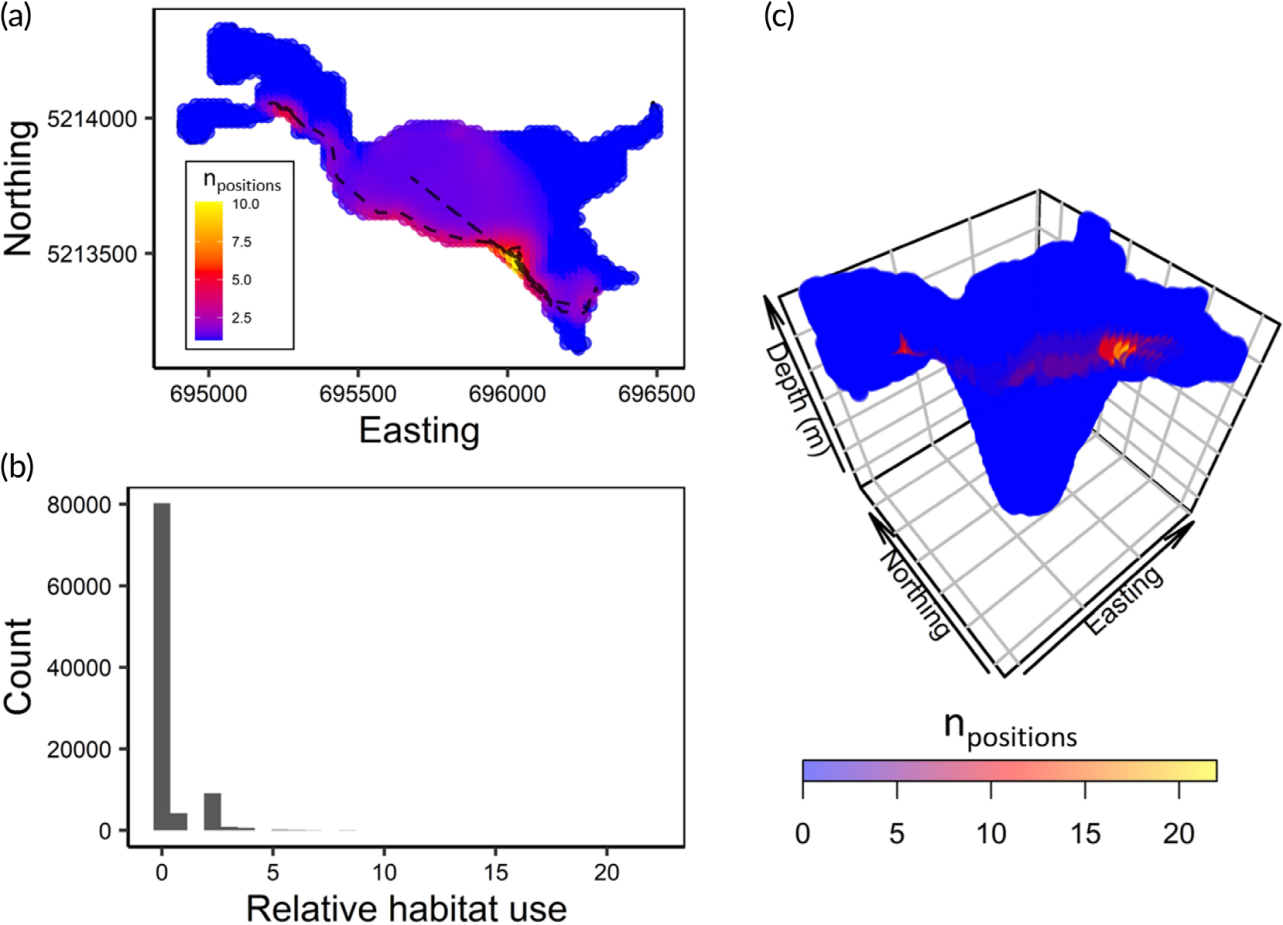
The daily relative habitat use of a white sucker (fish ID: 3543) on 2022‐08‐02 that is based on the frequency at which habitat voxels were included in the potential path volume (PPV) of individual path segments. Panel (a) presents the habitat voxels included in the daily PPV with color indicating the mean relative habitat use across the habitat voxels at each spatial coordinate. The dashed line represents the daily path of the fish. Panel (b) is the distribution of relative habitat use across all habitat voxels included in the daily PPV. Panel (c) is a three‐dimensional view of the daily PPV habitat voxels with color representing the number of times the habitat voxel is included in individual path segment PPVs.

In total, there were 480,447 white sucker positions in Turkey Lake between December 6, 2021, and October 24, 2022, after positions were removed based on the filtering criteria (Figure S4). There were 1957 unique fish‐date combinations at the daily temporal scale, with a mean of 234.9 positions per fish per day (median: 249 positions per fish per day). Among the nine tagged white sucker retained for analysis, four fish had around 72 to 162 days of positional data that included multiple successive positions that did not exceed the maximum time interval (1080 s) and were subsequently used in the analysis. The other five fish had more than 300 days of positional data available for analysis. Daily habitat volume estimates were calculated using the PPV models and ranged from 227 to 5,076,105 m^3^, with a mean habitat volume of 151,106 m^3^ (median: 64,694 m^3^), which is <2% of the total lake volume (see Figure 2 for daily habitat volume comparison among estimation methods). Although we did not conduct a thorough statistical analysis for the relative habitat use modification to the PPV model, we demonstrate how this approach can be used in future studies (Figure 3). From the daily habitat volume example (fish ID 3543 on 2022‐08‐02), 215 path segments and 95,628 habitat voxels were used to calculate habitat volume. Approximately 84% of the habitat voxels were not included in any path segment PPVs, and less than 1% of habitat voxels were included in five or more path segment PPVs (Figure 3b). Habitat voxels were included in 0% to 9.7% of the path segment PPVs.

**FIGURE 4:**
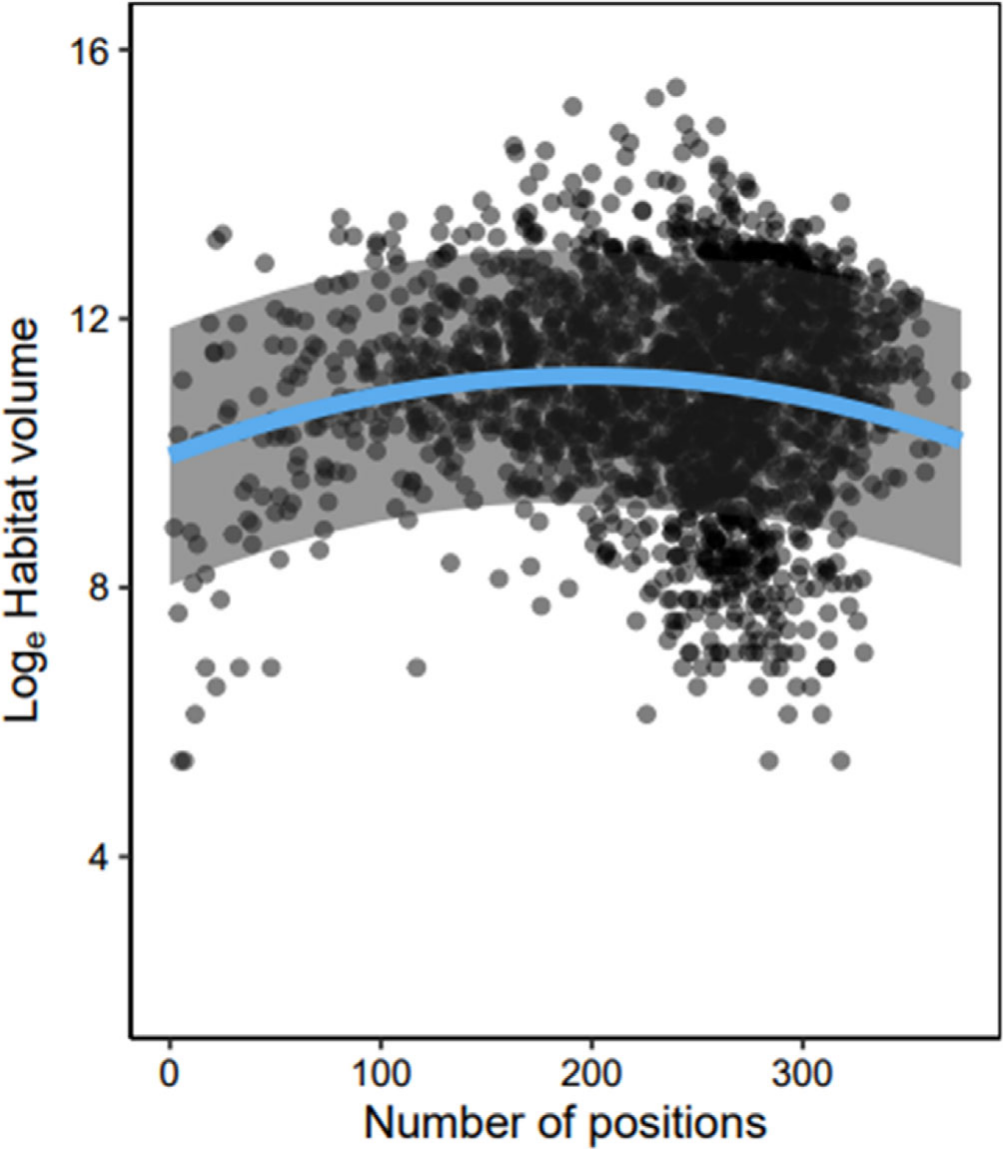
Relationship between the number of spatial positions and the estimated habitat volume (log_e_) from daily potential path volume (PPV) models (*n* = 1957). The blue line represents the fitted linear mixed model between habitat volume and the number of detections with both linear and quadratic terms ($R_{\text{cond}}^{2}$ = 0.109).

**FIGURE 5:**
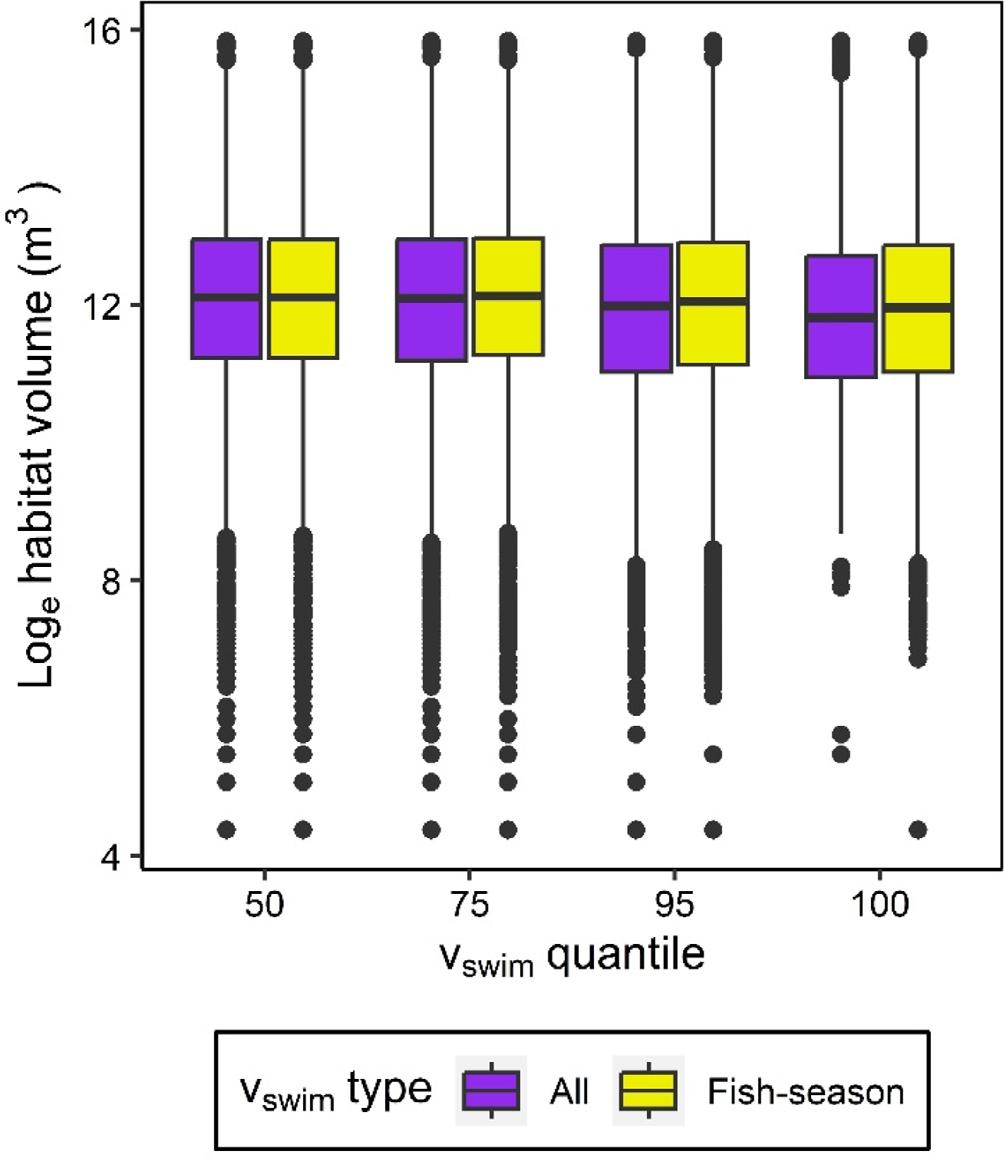
Daily habitat volume estimates across v_swim_ quantile values (50th, 75th, 95th, and 100th) and v_swim_ selection method (all vs. fish‐season). The “all” habitat volume estimates were obtained using the entire distribution of daily movement speeds, whereas the “season‐specific” daily habitat volume estimates were obtained using v_swim_ values that were specific to every possible fish‐season combination. The middle line in each box plot represents the median habitat volume value, with the boxes representing the interquartile ranges (IQR) for each distribution. The top and bottom of the boxes are the first and third quartiles (Q1 and Q3), respectively. The whiskers extend from Q1 and Q3 to the smallest and largest values, respectively, with a maximum length of 1.5 * IQR. Outliers outside of the whiskers are shown with black points.

The best model between PPV habitat volume estimates and the number of positions included both linear and quadratic terms, as this model had a better fit relative to the model with only the linear term and the null model (Table 1). Based on the values of the model parameters (Table 2), habitat volume initially increased with the number of positions but then began to decrease when there were many positions included in the model (Figure 4). Although the full model had the best fit, the model explained a small amount of variation in log_e_ habitat volume estimates (R^2^_marginal_ = 0.042, R^2^_conditional_ = 0.109). The parameterization of v_swim_ (quantile value and method) did slightly improve model fit to the data for habitat volume estimates relative to the intercept‐only null model based on AIC values (AIC_vswim_ = 63,009.1; AIC_null_ = 63,013.3; Figure 5); however, the difference was minimal (Δ AIC = 4.1). Therefore, we did not find considerable support that the parameterization of v_swim_ had a strong effect on the PPV habitat volume estimates for white sucker in our study lake.

**TABLE 1:** Comparison of linear mixed‐effects models between log_e_ habitat volume estimates and the number of spatial positions (n_positions_) included in the PPV models.

Model	AIC	ΔAIC	$R_{\text{marginal}}^{2}$	$R_{\text{conditional}}^{2}$
Intercept‐only	7461.56	60.81	0	0.038
n_positions_	7450.69	49.94	0.008	0.057
n_positions_ + n_positions_^2^	7400.75	0	0.042	0.109

*Note*: AIC and ΔAIC were used to evaluate the model goodness of fit. The $R_{\text{marginal}}^{2}$ and $R_{\text{conditional}}^{2}$ values indicate the variance explained in log_e_ habitat volume by the fixed and random effects.

Abbreviation: PPV, potential path volume.

**TABLE 2:** Model coefficients for best linear mixed‐effects model between habitat volume (log_e_ transformed) and the number of positions included in the PPV model.

Term	Estimate ± std. error	*df*	*t*‐Value
Intercept	10.184 ± 0.256	98.241	39.841*
n_positions_	0.013 ± 0.002	2044.800	6.126*
n_positions_^2^	‐3e‐05 ± 5.487e‐06	2034.387	−7.288*

*Note*: Significant *p*‐values (<0.001) are denoted with*.

Abbreviation: PPV, potential path volume.

**FIGURE 6:**
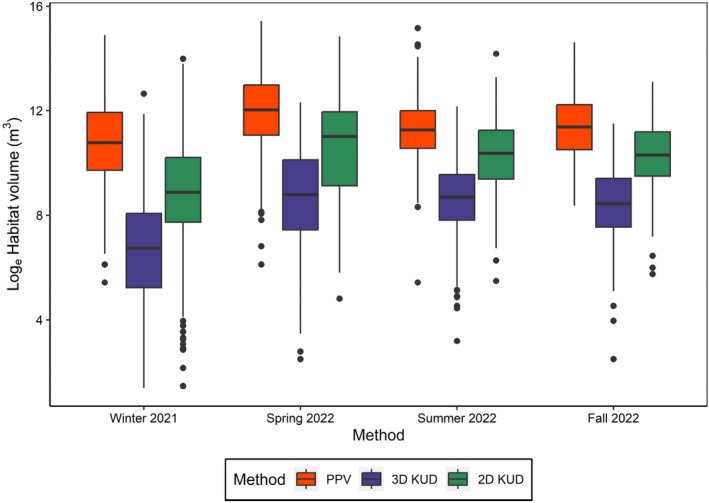
Habitat volume estimate (log_e_ transformed) distributions across seasons and estimation methods. Habitat volume estimates were significantly different across all season‐estimation method combinations (*p* < 0.05). The middle line in each box plot represents the median habitat volume value, with the boxes representing the interquartile ranges (IQR) for each distribution. The top and bottom of the boxes are the first and third quartiles (Q1 and Q3), respectively. The whiskers extend from Q1 and Q3 to the smallest and largest values, respectively, with a maximum length of 1.5 * IQR. Outliers outside of the whiskers are shown with black points.

The full fish habitat volume model, which included method, season, and method:season, had the most support with the lowest AIC value (Table 3) and was therefore considered the best model. PPV‐based habitat volume estimates were significantly greater than those from the 3D‐ and 2D‐KUD approaches although the difference between the PPV and 2D‐KUD was much smaller (Figure 6; Table S3). In general, the 3D‐KUD approach resulted in lower habitat volume estimates relative to the PPV and 2D‐KUD methods. Winter habitat volume estimates were significantly lower than those of the other three seasons. White sucker habitat volume was greater during the spring than the other three seasons and similar between the summer and fall. The best model had a marginal R^2^ value of 0.483 and a conditional R^2^ value of 0.538.

**TABLE 3:** Comparison of habitat volume (log_e_) linear mixed‐effects models with estimation method, season, and their interaction as potential fixed effects.

Model	n_terms_	AIC	ΔAIC	$R_{\text{marginal}}^{2}$	$R_{\text{conditional}}^{2}$
Method + season + method:season	3	22,320.622	0.000	0.499	0.547
Method + season	2	22,486.278	165.656	0.486	0.533
Method	1	23,290.723	970.101	0.399	0.468
Season	1	26,127.100	2806.478	0.084	0.130
Intercept—only	0	26,571.582	4250.960	0.000	0.067

*Note*: AIC and ΔAIC were used to evaluate the model goodness of fit. The $R_{\text{marginal}}^{2}$ and $R_{\text{conditional}}^{2}$ values indicate the variance explained in log_e_ habitat volume by the fixed and random effects.

**FIGURE 7:**
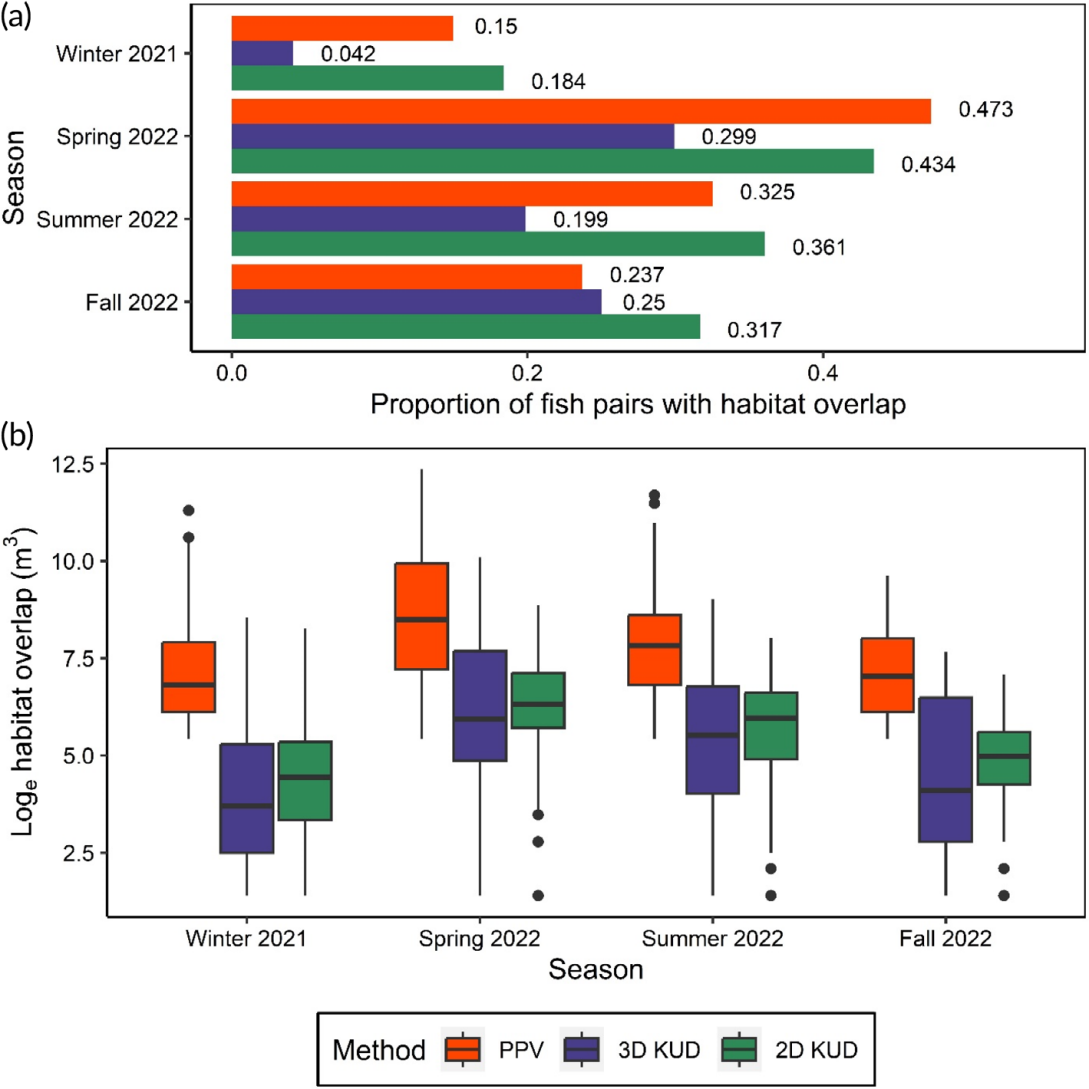
The proportion of fish pairs with overlapping habitat (a) and the distribution of habitat overlap estimates for fish pairs (b) across seasons and estimation methods. Fish pairs with habitats that did not overlap were excluded from panel (b). Color represents the estimation method (2D KUD, 3D KUD, and PPV [potential path volume]) used to calculate fish habitat volume.

There were 5927 unique daily white sucker pairs across all four seasons that were used to assess habitat overlap among individual tagged white sucker pairs. We found that season and the estimation method used to calculate habitat volume had a significant effect on the presence of daily habitat overlap for fish pairs relative to the null model (AIC_season+method_ = 11,844.7; AIC_null_ = 13,148.5). Across seasons, habitat overlap was greatest during the spring and lowest in the winter (Figure 7a; Table S2). For the PPV‐based habitat overlap estimates, 43.4% of the daily white sucker pairs had overlapping habitat in spring, whereas only 18.4% of pairs had habitat that overlapped during the winter. Habitat overlap differed across the estimation method considered with the presence of habitat overlap being less likely for the 3D‐KUD method relative to the 2D KUD and PPV (Figure 7a; Table S2). The magnitude of habitat overlap, when excluding daily fish pairs with no habitat overlap, was significantly influenced by season and estimation method relative to the null model (AIC_season+method_ = 10,393.6; AIC_null_ = 12,115.6). Seasonal trends in fish habitat overlap were similar across estimation methods with habitat overlap being the lowest in the winter, peaking in the spring and then gradually decreasing across the summer and fall (Figure 7b). When habitat overlap was present, the PPV method produced the largest habitat overlap estimates followed by the 2D‐KUD and 3D‐KUD estimates (Table S2).

## 4 DISCUSSION

In this study, we present the PPV model as a promising analytical tool to investigate the habitat use of aquatic organisms from a three‐dimensional perspective. Using telemetry data collected from white sucker in a northern temperate lake as a model system, the number of positions had an effect on PPV habitat volume estimates while the parameterization of v_swim_ did not. In a comparison to commonly used 3D‐ and 2D‐KUD methods for evaluating volumetric habitat use, we found that all methods detected seasonal differences in white sucker habitat volume, although habitat volume estimates differed among methods. Similar to the KUD‐focused approaches, the PPV model can be used to understand and explain patterns of how environmental characteristics dictate fish habitat use. We showcase the potential application of the PPV model to assess relative habitat use of fish across the study system during an annual cycle. We also used PPV models to assess habitat overlap among fish pairs and found that there were shifts in habitat overlap among fish pairs across the seasons. Most habitat overlap among pairs of white sucker occurred during the spring and summer, which corresponds to the periods with higher habitat volumes and with fewer fish pairs using the same habitat in the fall and winter when habitat volume was lower. The PPV model can be extremely valuable to both management and conservation as it provides valuable insights into fish habitat use by incorporating movement behavior within the context of a species' three‐dimensional habitat.

We found that the number of daily spatial positions used in the PPV model parameterization had a quadratic effect with intermediate numbers of spatial positions resulting in larger daily PPV habitat volume estimates relative to low or high numbers of spatial positions. This result aligned with our general expectation that fewer spatial positions would lead to smaller PPV habitat volume estimates because of a potential lack of spatial coverage from small sample sizes. On the contrary, a large number of spatial positions means that the time interval between spatial positions is smaller, leading to smaller path segment PPV estimates that contribute to the overall daily PPV estimates. Although the linear and quadratic terms significantly improved model fit, the model had low R^2^ values, both conditional and marginal. These low R^2^ values could suggest that the variation in the daily PPV space use estimates could be explained by additional habitat characteristics that were not considered in our study (e.g., availability of optimal thermal habitat and the spatial distribution of available resources). Future studies may consider investigating the relationship between the number of spatial positions and PPV habitat volume by incorporating covariates as well as examining these relationships in other types of systems (i.e., terrestrial and marine).

The PPV model is relatively simple to parameterize as the only major parameter to consider is the swim/movement speed of the organism (v_swim_), but habitat volume estimates can be highly sensitive to the v_swim_ parameter as this dictates the potential volume that an organism can move through between observed locations (Long & Nelson, 2012). We used the average daily movement speed for the v_swim_ parameter as this is more likely representative of a consistent speed at which tagged fish move in the wild and we were interested in the daily habitat use. Interestingly, we found that the v_swim_ parameter values used in our study did not have an effect on habitat volume estimates for white sucker in Turkey Lake, which contrasts the importance of the parameter in previous studies (Demšar & Long, 2019; Long & Nelson, 2012). The parameterization method, constant or fish‐season‐specific v_swim_ values, did not affect habitat volume estimates in our study, which could reflect a lack of variation in seasonal movement speeds among the tagged white sucker. The lack of an effect of v_swim_ on habitat volume may be directly attributed to the narrow range of v_swim_ values considered and to the spatial and vertical filtering of the habitat volume due to the study system (i.e., lake) considered. The finite habitat volume available in a confined lake ecosystem and the filtering would inhibit the expansion of the habitat volume when larger v_swim_ parameter values are considered in model development. The same reasoning could explain the effect of the number of observed positions on PPV habitat volume estimates. Similar to the findings of previous studies (Demšar & Long, 2019; Long & Nelson, 2012), we would expect that changes in the v_swim_ parameter would directly result in differences in PPV habitat volume estimates when a broader range of v_swim_ values and different sampling intervals for spatial positions are considered.

We compared the PPV models to KUD‐based approaches that evaluate animal habitat use; however, the application of these two approaches will likely be study‐specific. The kernel‐based methods excel at quantifying the density of habitat use across the study area to evaluate the home range and core areas of animal spatial occupancy (e.g., Dahl & Patterson III, 2020; Guzzo et al., 2016). Kernel‐density estimation can incorporate data from multiple individuals to develop habitat use models at higher organizational scales (e.g., population and assemblages) (Watson et al., 2019). PPV and other path‐focused analyses are generally parameterized for individual animals (Munaweera et al., 2021), although they can be readily applied to higher organizational levels, such as populations, through the aggregation of paths from multiple individuals or using hierarchical modeling to infer habitat use and movement behavior. The value of the PPV approach is that these models consider how animal movement influences habitat use and maintain the temporal structure of the animal movement. Incorporating animal movement in the PPV model ensures that paths connecting “hotspots”—those habitats with a high density of spatial positions—are incorporated into the habitat volume estimate. One disadvantage of the PPV model was that it could not be deployed to assess relative habitat use; however, our modification to the PPV model solves this issue by calculating the number of times that a habitat voxel is included in path segment PPVs within a given day. Although KUD‐based approaches have the potential to assess relative habitat use by using the KUD density estimates at each voxel, researchers often focus on habitat use at specific isopleths such as home range (95%) and core area (50%) to describe habitat use (Bloomfield et al., 2022; Guzzo et al., 2016). One caveat associated with the kernel‐density approach is that the observed detections are considered independent observations, even though the spatial and temporal autocorrelation associated with animal locations can result in biased habitat use estimates (Kraft et al., 2023). As the PPV model is a path‐based approach, the spatial and temporal structures of the telemetry data are inherently incorporated into habitat volume estimates.

Habitat volume estimates were not consistent across the three modeling techniques considered. The 2D‐KUD method, which assumes the entire lake depth, overestimated space use volume and overlap relative to the 3D‐KUD approach. The 2D‐KUD method was used to reflect using only data on lake bathymetry and the spatial locations of fish to estimate space use. We expected this method to overestimate habitat volume as fish typically do not use the entire water column available in most situations (Simpfendorfer et al., 2012). As for the PPV method, greater space use volumes were found compared to the KUD methods, perhaps because incorporating the potential habitat used by the animal rather than solely focusing on the observed habitat use from the spatial positions provides a larger range of outcomes. Interestingly, we did not expect white sucker to have extensive vertical movements, particularly to the same extent as species that exhibit daily vertical movement patterns or diving behavior, such as walleye (*Sander vitreus*) (Elliott et al., 2023), as white sucker are known to be associated with the lake bottom as benthivores (Logan et al., 1991; Richter et al., in review). Greater habitat volume from the PPV method, relative to the 3D‐KUD approach, could be due to the sampling interval and discontinuities among positions in the telemetry data collected. The sampling interval of positional data influences the resulting habitat volume estimates in PPV (Demšar & Long, 2019). Demšar and Long (2019) investigated the effects of sampling interval length on bird habitat volume and found that PPV size was positively associated with sampling interval duration. Long sampling intervals result in more uncertainty associated with animal movement (Rowcliffe et al., 2012) and an increase in the potential habitat the animal can use within that period. The larger potential habitat volume estimates would then be similar to those obtained from the 2D‐KUD that would tend to overestimate habitat volume itself as it assumes that the fish use the entire depth profile of the lake. Future studies could evaluate whether finer sampling intervals (< 240–360 s maximum intervals) are needed for the application of PPV models to analyse empirical telemetry data of aquatic animals.

Habitat overlap is a focal point of animal space use studies (e.g., Bertolino et al., 2013; Madigan et al., 2022) and can be evaluated using PPV models (Demšar & Long, 2019). Our results showed that the number of fish pairs with daily habitat overlap was season dependent. The observed greater habitat overlap among fish pairs during the springs coincides with the spawning period for white sucker (Corbett & Powles, 1983; Jones & Mackereth, 2016) when they congregate in large numbers in shallow waters with gravel substrate. As for the winter, habitat overlap among individuals was at its lowest relative to the other seasons. Temperature‐related changes in fish metabolic cost could result in lower daily habitat volume (McMeans et al., 2020) and, subsequently, reduce the potential for habitat overlap among fish pairs during the winter. Interestingly, similar daily movement rates were observed by the Turkey Lake white sucker population across all seasons (Richter et al., in review); thus the seasonal changes in habitat overlap among individuals could suggest possible seasonal changes in social or foraging behavior. For example, lake trout in a small boreal lake were observed to occupy much smaller areas in winter compared to summer, but daily movement rates were similar between seasons (Blanchfield et al., 2009). Although little is known about the social behavior of adult white sucker, specifically during the winter, we would expect that adult white sucker would congregate in small groups throughout the year, similar to other catostomid species (Billman, 299) and white sucker juveniles (Krause et al., 1998). The application of these habitat modeling techniques can be improved by incorporating life‐history details to help explain empirical patterns of animal habitat use and can be used to further our understanding of the social behavior of species across seasons.

The data and information collected in telemetry studies can help inform key management and conservation efforts regarding habitat use (Bendall et al., 2012; Binder et al., 2018). For example, telemetry data have been used to develop conservation strategies in the New York Bight for Atlantic sturgeon (*Acipenser oxyrinchus*) (Melnychuk et al., 2017), to understand how fish species use marine protected areas (Novak et al., 2020), and to document fisheries interactions with shortfin mako sharks (*Isurus oxyrinchus*) (Byrne et al., 2017). These examples have focused on the spatial component of telemetry data with limited consideration of the vertical movement of aquatic organisms. The incorporation of three‐dimensional data can support well‐informed decisions in management with regard to fish habitat use, especially for cool‐ and cold‐water species in temperate lakes that are susceptible to seasonal changes in their thermal‐depth profile (Bloomfield et al., 2022; Rodrigues et al., 2022).

In our study, we examine the application of the PPV model to evaluate animal space use patterns in a three‐dimensional context. The PPV and KUD models can both serve as suitable analytical tools that can be used to evaluate three‐dimensional habitat use, and their applications are likely to be dependent on the objectives of the project. The PPV model incorporates movement and the amount of potential space use by animals as they move throughout their habitat and can provide insight into habitat use patterns that could otherwise be missed when focusing only on the observed telemetry positions of the animals. Overestimation of habitat use estimates can lead to poor allocation of resources in conservation and management efforts. On the contrary, underestimating animal habitat use may result in excluding essential habitats. We showcase the PPV modeling approach for a freshwater fish species in a small temperate lake; however, more research is necessary to further our understanding of how these methods can be readily applied to biota in other aquatic ecosystems and how this approach may differ from commonly used telemetry‐focused modeling techniques.

## AUTHOR CONTRIBUTIONS

Paul J. Blanchfield and Karen E. Smokorowski conceived the whole‐ecosystem research programme. All authors contributed to the data collection. Ian A. Richter and Paul J. Blanchfield contributed to the conception and design of the study. Ian A. Richter contributed to the primary writing and data analysis. Paul J. Blanchfield and Karen E. Smokorowski provided support for the methodology, interpretation of the results, and the writing of the manuscript (review and editing).

## Supporting information


**Data S1.** Supporting information.

## Data Availability

The data collected by Fisheries and Oceans Canada are available upon request. Please contact the senior author (paul.blanchfield@dfo-mpo.gc.ca) for details about the code or data used in this study.
