# Supplementary material for: Incorporating vertical movement of fishes in habitat use models
Source: J Fish Biol. 2024 Jul 9;106(5):1570–84. doi: 10.1111/jfb.15857 (PMC12120347; doi:10.1111/jfb.15857)
Supplement: Supplementary file 1 — Data S1. Supporting information. [file JFB-106-1570-s001.docx]

Table S1. Specifications for the 69 kHz acoustic transmitters that were surgically implanted in the white sucker fish in Turkey Lake. Specifications were obtained from Innovasea Systems Inc. The weight values provided are the weight of the transmitter in the water and the pressure resolution values are based on a maximum depth of 34 m and room temperature.

| Transmitter Model | Diameter (mm) | Length (mm) | Weight (g) | | Detection interval range (sec) | Pressure resolution (m) |
| --- | --- | --- | --- | --- | --- | --- |
| V9P-2x | 7 | 31 | 2.8 | [240, 360] | | 0.15 |
| V13P-2x | 13 | 39 | 5.5 | [240, 360] | | 0.15 |

Table S2. Details of the nine white sucker fish tagged and tracked during the study period.

| **Fish ID** | **Transmitter model** | **Floy Tag** | **Total length(mm)** | **Weight (g)** | **Surgery date** |
| --- | --- | --- | --- | --- | --- |
| 3538 | V13P-2x | 8836 | 425 | 890 | 2021-10-27 |
| 3539 | V13P-2x | 10195 | 525 | 1500 | 2021-10-27 |
| 3541 | V13P-2x | 10196 | 527 | 1560 | 2021-10-27 |
| 3542 | V13P-2x | 10198 | 444 | 1390 | 2021-10-27 |
| 3543 | V13P-2x | 10197 | 465 | 1480 | 2021-10-27 |
| 3544 | V13P-2x | 10199 | 475 | 1450 | 2021-10-27 |
| 14659 | V9P-2x | 8837 | 535 | 1700 | 2021-10-28 |
| 14662 | V9P-2x | 8832 | 360 | 610 | 2021-10-27 |
| 14663 | V9P-2x | 8833 | 404 | 730 | 2021-10-27 |
|  |  |  |  |  |  |

Table S3. Model coefficients for the linear mixed effects model that relates estimation method (i.e., PPV, 3D-KUD, and 2D-KUD), season (i.e., winter, spring, summer, and fall), and the interaction between the two variables to daily white sucker habitat volume (log_e_ m^3^). Individual fish was included as a random effect.

| Term | Estimate ± Std. Error | df | t-value (Pr(>\|t\|)) |
| --- | --- | --- | --- |
| (Intercept) | 10.60 ± 0.17 | 9.99 | 62.01 (< 0.01) |
| Method – 3D KUD | -3.98 ± 0.07 | 5950.02 | -58.75 (< 0.01) |
| Method – 2D KUD | -1.01 ± 0.07 | 5950.02 | -14.97 (< 0.01) |
| Season - Spring | 0.99 ± 0.14 | 5955.10 | 6.95 (< 0.01) |
| Season - Summer | 0.68 ± 0.09 | 5955.79 | 7.93 (< 0.01) |
| Season - Fall | 0.78 ± 0.15 | 5955.52 | 5.16 (< 0.01) |
| 3D-KUD:Spring | 1.06 ± 0.20 | 5949.91 | 5.26 (< 0.01) |
| 2D-KUD:Spring | 0.66 ± 0.20 | 5949.91 | 3.28 (< 0.01) |
| 3D-KUD:Summer | 1.35 ± 0.12 | 5949.93 | 11.34 (< 0.01) |
| 2D-KUD:Summer | 0.83 ± 0.12 | 5949.93 | 6.92 (< 0.01) |
| 3D-KUD:Fall | 1.00 ± 0.21 | 5949.91 | 4.77 (< 0.01) |
| 2D-KUD:Fall | 0.65 ± 0.21 | 5949.91 | 3.10 (< 0.01) |

Table S4. Details for the two-part fish habitat overlap model that describes the habitat overlap among white sucker pairs using season and method as predictor variables. The first model was a mixed-effects logistic regression with habitat overlap as a binary response variable. The second model was a linear mixed-effects model that only considered nonzero values of habitat overlap. The intercept term is representative of the effects of winter (season) and 2D KUD (method) on habitat overlap for both models.

| Model #1 – Binary response variable in mixed-effects logistic regression | | | |
| --- | --- | --- | --- |
| Term | **Estimate ± Std. Error** | **z-value (Pr(>\|z\|))** | |
| (Intercept) | -1.81 ± 0.17 | -10.69 (< 0.01) | |
| Method – 3D KUD | -1.42 ± 0.07 | -21.37 (< 0.01) | |
| Method – PPV | -0.24 ± 0.05 | -4.65 (< 0.01) | |
| Season - Spring | 2.13 ± 0.10 | 21.70 (< 0.01) | |
| Season - Summer | 1.55 ± 0.07 | 21.79 (< 0.01) | |
| Season - Fall | 1.11 ± 0.15 | 7.61 (< 0.01) | |
| Model #2 – Continuous response variable in a linear mixed-effects model | | | |
| Term | **Estimate ± Std. Error** | **df** | **t-value (Pr(>\|t\|))** |
| (Intercept) | 4.27 ± 0.06 | 74.37 | 72.37 (< 0.01) |
| Method – 3D KUD | -0.24 ± 0.08 | 2852.71 | -2.95 (0.03) |
| Method – PPV | 2.45 ± 0.06 | 2852.35 | 40.47 (< 0.01) |
| Season - Spring | 1.81 ± 0.09 | 5955.09 | 19.03 (< 0.01) |
| Season - Summer | 1.10 ± 0.07 | 5955.79 | 14.75 (< 0.01) |
| Season - Fall | 0.340 ± 0.16 | 5955.52 | 2.12 (< 0.01) |


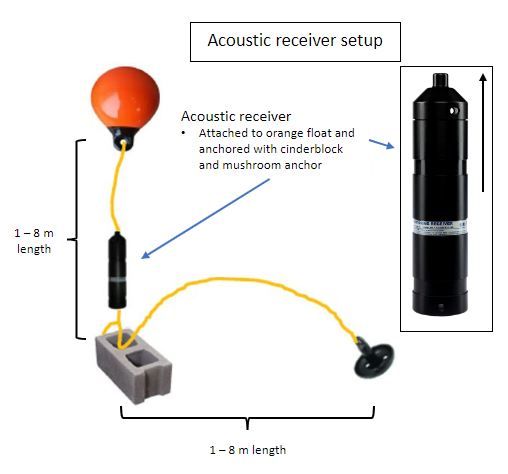


Figure S1. Diagram of the acoustic receiver deployment (n = 29) for the fine-scale acoustic telemetry system deployed in Turkey Lake, ON. The position of the orange float was below the water surface to ensure the polysteel was taut and the consistent upwards positioning of the acoustic receiver. The acoustic receiver was positioned 1 – 4 m above the cinder block anchor.


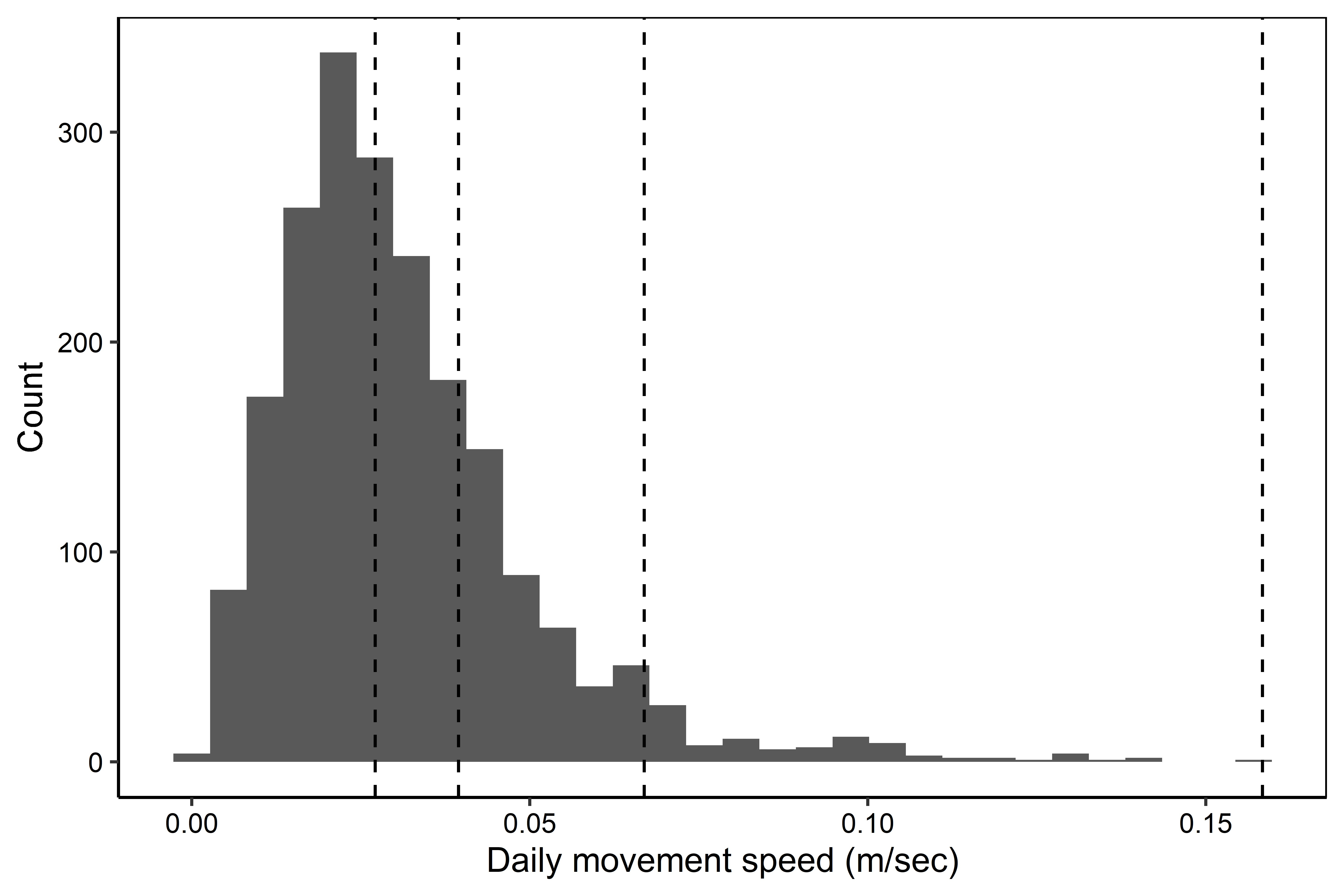


Figure S2. Distribution of the daily movement speeds for acoustically tagged white sucker (n = 9) in Turkey Lake (Canada) across the study period (~1 y). Daily movement speed was calculated as the sum of the distance moved in a day (based on telemetry positions) divided by the time difference between the first to last observations for a given day. The dashed lines in the distribution represent Q50, Q75, Q95, and Q100 (i.e., maximum) of the daily movement speed distribution from left to right which were used to estimate the v_max_ parameter in the PPV models.





Figure S3. Distribution of the daily movement speeds for all possible season-fish ID combinations (n = 25). Daily movement speed was calculated using telemetry positions as the sum of the distance travelled in a day divided by the time difference from the first to last observations for the given day. The dashed lines in the distribution represent Q50, Q75, Q95, and Q100 (i.e., maximum) of the daily movement speed distribution from left to right were used to estimate the v_max_ parameter in the PPV models.


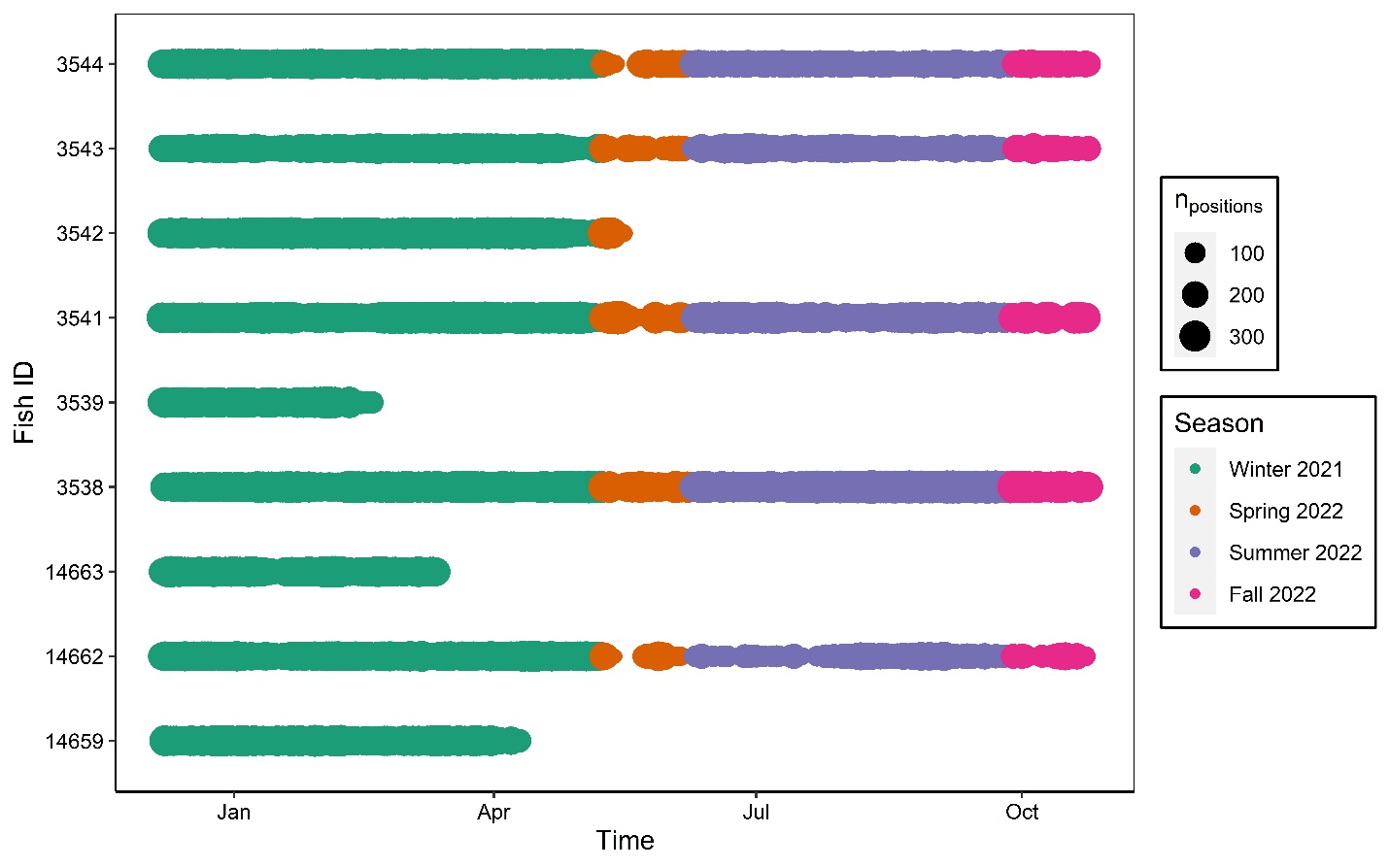


Figure S4. Available daily positional data collected from the fine-scale acoustic telemetry system deployed in Turkey Lake, ON, for each tagged white sucker (n = 9) across the study period (December 6^th^, 2021 to October 24^th^, 2022).
